# S100A4 promotes pancreatic cancer progression through a dual signaling pathway mediated by Src and focal adhesion kinase

**DOI:** 10.1038/srep08453

**Published:** 2015-02-13

**Authors:** Pulin Che, Youfeng Yang, Xiaosi Han, Meng Hu, Jeffery C. Sellers, Angelina I. Londono-Joshi, Guo-Qiang Cai, Donald J. Buchsbaum, John D. Christein, Qinjiu Tang, Dongquan Chen, Qianjun Li, William E. Grizzle, Yin Ying Lu, Qiang Ding

**Affiliations:** 1 Department of Medicine, University of Alabama at Birmingham, Birmingham, Alabama, USA; 2 Department of Pathology, University of Alabama at Birmingham, Birmingham, Alabama, USA; 3 Department of Neurology, University of Alabama at Birmingham, Birmingham, Alabama, USA; 4 Department of Radiation Oncology, University of Alabama at Birmingham, Birmingham, Alabama, USA; 5 Department of Surgery, University of Alabama at Birmingham, Birmingham, Alabama, USA; 6 Institute of Edible Fungi, Shanghai, China; 7 Center of Therapeutic Research for Hepatocellular Carcinoma, 302 hospital, Beijing, China

## Abstract

S100A4 expression is associated with poor clinical outcomes of patients with pancreatic cancer. The effects of loss or gain of S100A4 were examined in pancreatic cancer cell lines. S100A4 downregulation remarkably reduces cell migration and invasion, inhibits proliferation, and induces apoptosis in pancreatic tumor cells. S100A4 downregulation results in significant cell growth inhibition and apoptosis in response to TGF-β1, supporting a non-canonical role of S100A4 in pancreatic cancer. The role of S100A4 in tumor progression was studied by using an orthotopic human pancreatic cancer xenograft mouse model. Tumor mass is remarkably decreased in animals injected with S100A4-deficient pancreatic tumor cells. P27^Kip1^ expression and cleaved caspase-3 are increased, while cyclin E expression is decreased, in S100A4-deficient pancreatic tumors *in vivo*. S100A4-deficient tumors have lower expression of vascular endothelial growth factor, suggesting reduced angiogenesis. Biochemical assays revealed that S100A4 activates Src and focal adhesion kinase (FAK) signaling events, and inhibition of both kinases is required to maximally block the tumorigenic potential of pancreatic cancer cells. These findings support that S100A4 plays an important role in pancreatic cancer progression *in vivo* and S100A4 promotes tumorigenic phenotypes of pancreatic cancer cells through the Src-FAK mediated dual signaling pathway.

S100A4, also known as metastasin (Mts1)^1^,^2^, belongs to the S100 family, and it is a calcium-binding protein with two EF-hands^3^,^4^,^5^. S100A4 is involved in a variety of physiological functions, such as cell motility, adhesion, proliferation, invasion, and metastasis^3^,^4^,^6^,^15^. S100A4 is considered as a mediator of tumor progression and metastasis^3^,^6^. S100A4 can suppress the BNIP3 expression and contributes to chemoresistance and survival in pancreatic cancer cells^16^. S100A4 is involved in epithelial mesenchymal transition mediated by the Shh-Gli1 signaling pathway^17^, and S100A4 promotes cell invasion in pancreatic cancer cells^18^. S100A4 is overexpressed in pancreatic cancer^7^, and is also frequently overexpressed in other metastatic cancers, including prostate^8^,^9^, ovarian^10^, and breast carcinomas^11^,^12^. Increased S100A4 expression has been strongly associated with poor clinical outcomes of the pancreatic cancer^7^,^13^,^14^. Although aberrant S100A4 expression is an independent biomarker of poor outcome, the molecular mechanisms by which S100A4 regulates pancreatic cancer progression *in vivo* are not completely understood. Whether S100A4 directly contributes to pancreatic cancer progression *in vivo* or is just a secondary effect of other changes during pancreatic cancer progression remains to be answered.

Focal adhesion kinase (FAK) is a non-receptor tyrosine kinase, that is upregulated in many types of cancers, including pancreatic cancers^19^,^20^. FAK is activated when its tyrosine-397 (Y397) is phosphorylated, and maximal FAK activation requires binding of Src kinase^21^,^22^. Increased FAK activation is positively associated with the stage and grade of pancreatic cancer^20^,^23^. FAK mediates cell migration through regulation of focal adhesion turnover, and cell proliferation and survival through downstream signaling proteins, such as mitogen-activated protein kinases, cyclins, phosphatidylinositide 3-kinases (PI3K), and Src kinase^22^,^24^,^25^,^26^,^27^,^28^. Src kinase plays important roles in cellular functions, such as cell motility and proliferation^29^. Increased Src activity has been reported in many types of tumors^30^, and approximately 60% of pancreatic cancers display increased Src activity which is associated with a poor prognosis^31^. Src kinase can be activated by growth factors and intracellular signaling proteins^32^. FAK also can activate Src kinase by releasing Src from its auto-inhibitory domain (Y531 of Src)^22^,^32^. As S100A4, FAK, and Src are all associated with poor prognosis in pancreatic cancer, it is important to understand whether they promote pancreatic cancer progression in a coordinated manner.

In this study, we examined the effects of S100A4 on the aggressive characteristics of pancreatic cancer cells *in vitro* by modulating S100A4 expression levels (loss and gain approaches), and pancreatic cancer growth *in vivo* by using an orthotopic human pancreatic cancer xenograft mouse model. The findings provide evidence that S100A4 facilitates pancreatic cancer progression through promoting cell migration and invasion, anchorage-independent growth, angiogenesis, and tumor survival. S100A4 also plays an important role in protecting pancreatic cancer cells against transforming growth factor beta (TGF-β)-induced growth inhibition and apoptosis. S100A4 enables intracellular FAK and Src signaling events that operate as a dual signaling pathway and underlie the tumorigenic potential of pancreatic carcinoma cells. Together, these results indicate that S100A4 would be an attractive therapeutic target in pancreatic cancer.

## Results

### S100A4 mediates pancreatic cancer cell migration, invasion, and anchorage-independent growth

S100A4 protein expression in human pancreatic tumor samples is more than that in non-tumor control samples as reported by others^7^,^13^. To study the role of S100A4 in pancreatic cancer progression, the effects of loss (and gain) of function of S100A4 on cell migration, invasion, and cell growth were examined. We established stable S100A4 knockdown cell lines by infecting human pancreatic cells S2VP10 and MIA PaCa-2 with lentiviruses encoding shRNA for S100A4, S100A4 shRNA #1 and #2, respectively. Both the mRNA and protein levels of S100A4 were reduced by more than 90% in these stable cell lines when compared with the scrambled control, respectively (Supplementary Figs. S1B–S1D).

**Figure 1:**
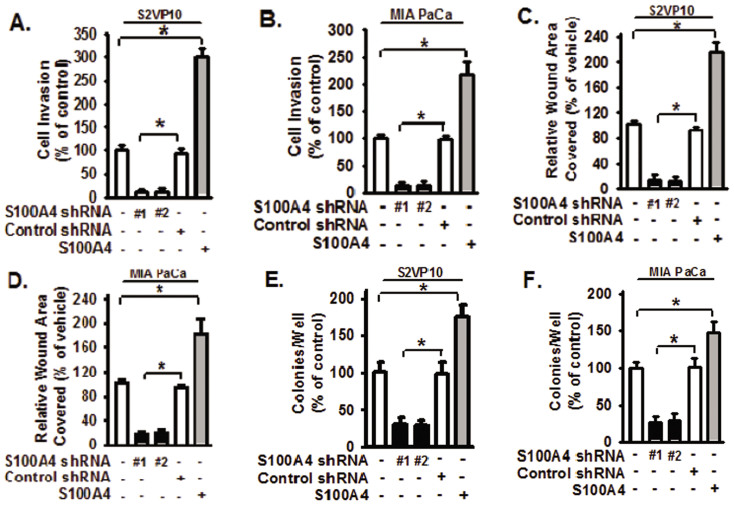
S100A4 promotes migration, invasion, and anchorage-independent growth of pancreatic tumor cell lines. The functional role of S100A4 on promoting the tumorigenic potential of pancreatic cancer cells was studied through loss (or gain) of S100A4 approach. Loss of S100A4 was achieved with lentiviral vectors as shown in Supplementary Figure S1. S2VP10 and MIA PaCa-2 (MIA PaCa) pancreatic cancer cells with S100A4 shRNA (#1 and #2) or control non-targeting shRNA were subjected to the following assays. (A–B) Cell invasion assays, (C–D) wound closure motility assays, and (E–F) anchorage-independent/soft-agar growth assays. Assays were performed as described in Methods. S2VP10 and MIA PaCa-2 (MIA PaCa) cells were also treated with or without S100A4 (1 μg/ml) and subjected to the same assays. Representative images of soft-agar growth assays are shown in supplementary Fig. S5. S2VP10 cells are shown in panels (A, C, and E). MIA PaCa cells are shown in panels (B, D, and F). The experiments were repeated 3–4 times and with 2–3 replicates. Data are represented as mean + SE. * represents p < 0.01 for the indicated two groups.

The silencing of S100A4 resulted in decreased cell invasion (Figs. 1A–B, bars 2 & 3 versus bar 1, p < 0.01), cell migration (Figs. 1C–D, bars 2 & 3 versus bar 1, p < 0.01), and soft agar colony formation (Figs. 1E–F, bars 2 &3 versus bar 1, p < 0.01), in both S2VP10 and MIA PaCa-2 stable cell lines. The silencing of S100A4 also resulted in decreased cell migration in Pan 2.03 and BxPC-3 pancreatic cancer cell lines (supplementary Fig. 5S, A–B, bars 2 & 3 versus bar 1, p < 0.01). Exogenous S100A4 treatment enhanced cell invasion and cell migration, and soft agar colony formation in S2VP10 and MIA PaCa-2 cells (Figs. 1A-F, bar 5 versus bar 1, respectively). Pancreatic cancer cells treated with scrambled control shRNA did not show significant difference in cell invasion, migration, and soft agar colony formation when compared to cells without treatment (Figs. 1A–F, bar 4 versus bar 1). In addition, cells treated with empty lentiviral vectors showed no difference relative to cells without treatment (data not shown). These findings support that S100A4 plays an important role in pancreatic cancer cell invasion, migration, and anchorage-independent growth.

### S100A4 downregulation induces pancreatic cancer cell growth inhibition and apoptosis in response to transforming growth factor beta-1 (TGF-β1) treatment

**Figure 2:**
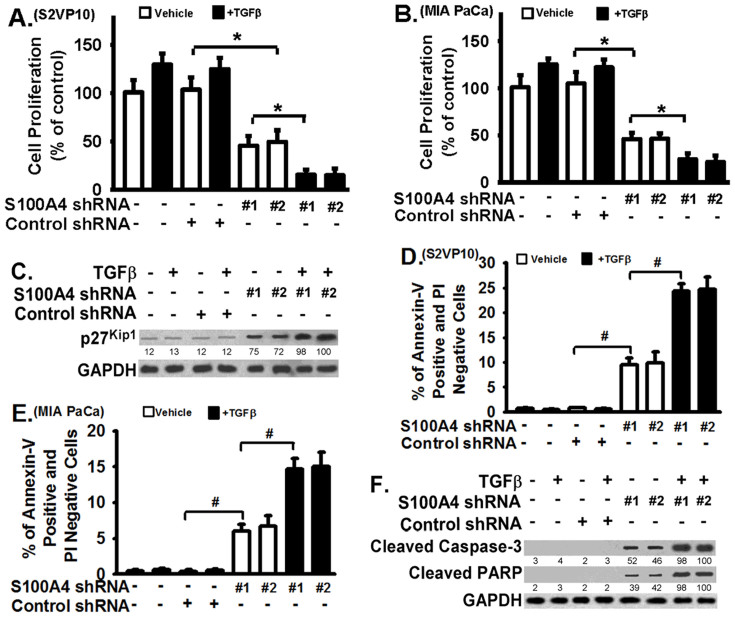
S100A4 downregulation sensitizes pancreatic tumor cell lines to TGF-β1-induced cell growth inhibition and apoptosis. S100A4 downregulation was achieved with lentiviral vectors as shown in Fig. 1. S2VP10 and MIA PaCa-2 (MIA PaCa) cells with or without shRNA infection were serum starved with serum-free medium (DMEM with 1% BSA) for 24 hours, followed by treatment with TGF-β1 (4 ng/ml) or vehicle for 24 hours in serum-free medium. (A–B) Cell proliferation assays were performed as described in Methods with S2VP10 and MIA PaCa-2 (MIA PaCa) cells, respectively. (C) S2VP10 cells infected with S100A4 or control shRNA were treated with or without TGF-β1 as described above. Cells were lysed, and equivalent amount of lysates were Western blotted with indicated antibodies for proliferation signaling event. Numbers below images represent densitometry of band intensity. (D–E) Annexin-V and PI labeling apoptosis assays were performed as described in Methods with S2VP10 and MIA PaCa-2 (MIA PaCa) cells, respectively. The percentage of early apoptotic cells were marked by Annexin-V-positive and PI-negative. (F) S2VP10 cells infected with S100A4 or control shRNA were treated without or with TGF-β1 as described above. Cells were lysed, and equivalent amount of lysates were Western blotted with indicated antibodies for apoptotic signaling events. The antibodies specifically recognize the cleaved Caspase-3 and PARP and do not recognize the full length forms. The experiments were repeated 3–4 times. Data are represented as mean + SE. * represents p < 0.01 and # represents p < 0.001 for the indicated two groups.

TGF-β1 plays an important role in tumor development via its anti-proliferative and other effects that reduce tumor aggressiveness; however, many tumor cells manage to ignore TGF-β1 or utilize TGF-β1 as a promoter for tumor progression. In S2VP10 and MIA PaCa-2 pancreatic cancer cells, TGF-β1 alone slightly increased cell growth (about 20%) (Figs. 2A and 2B, bar 2 versus bar 1, respectively), but did not affect the percentage of cells labeled annexin-V-positive and propidium iodide (PI)-negative (Figs. 2D and 2E, bar 2 versus bar 1, respectively), indicating that the phenotype of these pancreatic cancer cells is resistant to TGF-β1. Early apoptotic cells are considered as annexin-V-positive and PI-negative.

S100A4 downregulation sensitized pancreatic cancer cells to TGF-β1-mediated growth inhibition and apoptosis. While TGF-β1 had no effect on control cells (no treatment, or treated with non-targeting shRNAs), TGF-β1 remarkably inhibited cell growth (Figs. 2A and 2B, bars 7 & 8 versus bars 5 & 6 or bar 2) and increased the percentage of annexin-V-positive and PI-negative cells (Figs. 2D and 2E, bars 7 & 8 versus bars 5 & 6 or bar 2) in S100A4-downregulated S2VP10 and MIA PaCa-2 cells. S100A4 downregulation alone also reduced cell growth (Figs. 2A and 2B, bars 5 & 6 versus bar 3) and increased the percentage of annexin-V-positive and PI-negative cells (Figs. 2D and 2E, bars 5 & 6 versus bar 3) compared to controls cells. S100A4 downregulation increased the percentage of cells in G0/G1 phase, but decreases the percentage of cells in S phase (supplementary Figure S4), supporting that S100A4 plays a role in cell cycle progression in pancreatic tumor cells. The inhibitory effect of S100A4 downregulation on cell cycle progression was further enhanced by TGF-β1 treatment (supplementary Figure S4).

Cyclins (such as cyclin E) regulate cell cycle transition from G1 to S phase, and cyclin-dependent kinase inhibitor p27^Kip^ inhibits cyclin-mediated cell progression. p27^Kip^ has been shown to regulate breast cancer cell functions^33^ and cell cycle progression of glioblastoma cells^34^. Caspases play important roles in apoptosis, and both intrinsic and extrinsic apoptotic pathways induce activation of caspase-3 (cleavage of full length of caspase-3)^35^,^36^,^37^. S100A4 downregulation increased p27^Kip1^ expression (Fig. 2C, and supplementary Figure S7A), and induced cleaved caspase-3 and cleaved poly(ADP-ribose) polymerase (PARP) (Fig. 2F, and supplementary Figure S7B). TGF-β1 treatment enhanced the effects of S100A4 downregulation on p27^Kip1^ expression, cleaved caspase-3, and cleaved PARP, because the levels of these mediators were further increased in response to TGF-β1 (relative to controls) in these S100A4-deficient S2VP10 cells (Figs. 2C and 2F, supplementary Figure S7A and S7B, lanes 7 & 8 versus lanes 5 and 6, respectively). To further assess the contribution of caspase activation to the apoptotic effect of S100A4-downregulation and TGF-β1 treatment, the percentage of apoptotic cells were examined in cells treated with the pan-caspase inhibitor Z-VAD-fmk (20 μmol/L). Z-VAD-fmk treatment significantly reduced the percentage of annexin-V-positive and PI-negative cells (supplementary Fig. S5, D–E), supporting the role of caspase-mediated apoptosis during these events. A recent study shows that S100A4 promotes p53 degradation^38^ and the p53 homeostasis is important for tumorigenesis^39^. S100A4 downregulation leads to increased p53 expression and decreased Akt phosphorylation (at Ser473); but, the effect of S100A4 downregulation on p53 and Akt phosphorylation are not sensitized by TGF-β1 treatment (new supplementary Figure S3), suggesting that p27 and caspase-3 signaling are specifically sensitive to TGF-β1 treatment in addition to S100A4 downregulation. Taken together, Figure 2 demonstrates that S100A4 expression is important for the resistant phenotype of pancreatic cancer cells to TGF-β1-mediated growth inhibition and apoptosis.

### S100A4 plays an important role in pancreatic tumor growth *in vivo*

**Figure 3:**
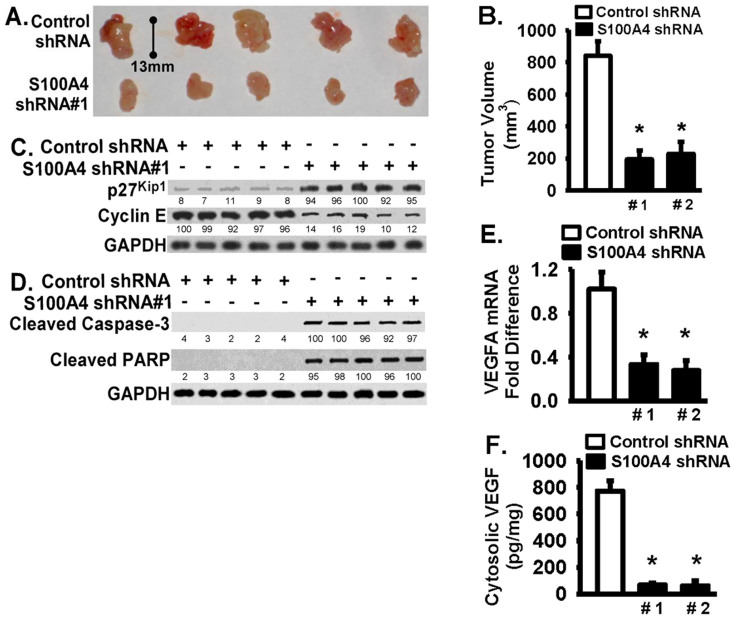
S100A4 downregulation significantly reduces pancreatic tumor growth, impairs expression of cyclin E and VEGFA, and increases p27^Kip1^ expression and levels of cleaved caspase-3 and PARP *in vivo* in pancreatic cancer mouse model. S2VP10 cells stably infected with S100A4 or control shRNA were used in a human pancreatic cancer orthotopic xenograft mouse model. Primary tumors in the pancreas were excised at day 21 post injection. (A) Representative tumor images (n = 5 animals, per group) are shown. Bottom panel shows the S100A4-deficient tumors by using S100A4 shRNA #1. (B) Tumor volumes were calculated as described in Methods. Black bar for the S100A4-deficient tumors (n = 5–6 animals, per bar). (C–D) Tumor tissues were lysed, and equivalent amount of whole tissue lysates were Western blotted with indicated antibodies. GAPDH was used as loading control. (E–F) Total RNA was extracted from tumor tissues and followed by quantitative RT-PCR to determine the mRNA level of vascular endothelial growth factor A (VEGFA) in panel (E). VEGF protein level was determined by ELISA assay in panel (F). The experiments were repeated 3–4 times. Data are represented as mean + SE. * represents p < 0.01 compared to the control shRNA group.

The *in vivo* requirements for S100A4 were tested by inoculating S2VP10 cells (expressing either S100A4 #1 or control shRNAs) into the tail of the pancreas of female SCID mice^40^. Mice in the control group developed palpable tumors at day 21 post injection and all animals had tumors (Fig. 3A, top panel). Mice injected with stable S100A4-downregulated cells showed a significant delay in tumor development (Fig. 3A, bottom panel). As shown in Fig. 3B, the mean size of S100A4-deficient tumors was about 4 fold less than the mean size of tumors in control animals (about 194 mm^3^ versus 823 mm^3^, p < 0.01). The S100A4-deficient tumors showed increased p27^Kip1^ expression and decreased cyclin E expression compared with the control tumors (Fig. 3C and Supplementary Fig. S2A). Increased cell cycle inhibitor (p27^Kip1^) and decreased cell growth signaling protein (cyclin E) suggest that the cellular proliferation pathways are impaired in S100A4-deficient tumors. The levels of cleaved caspase-3 and cleaved PARP were increased in S100A4-deficient tumors relative to the control tumors (Fig. 3D and Supplementary Fig. S2B), indicating increased apoptosis in S100A4-deficient tumors. Angiogenesis is critical for tumor growth, and vascular endothelial growth factor (VEGFA) is frequently expressed in many tumors^41^. The VEGFA mRNA and protein levels were significantly decreased in S100A4-deficient tumors compared to control tumors (Figs. 3, E–F), suggesting that VEGF-mediated angiogenesis could be limited or decreased in S100A4-deficient tumors.

### S100A4 mediates Src and FAK activation, and S100A4 downregulation reduces Src and FAK activation in pancreatic cancer cells *in vitro* and *in vivo*

**Figure 4:**
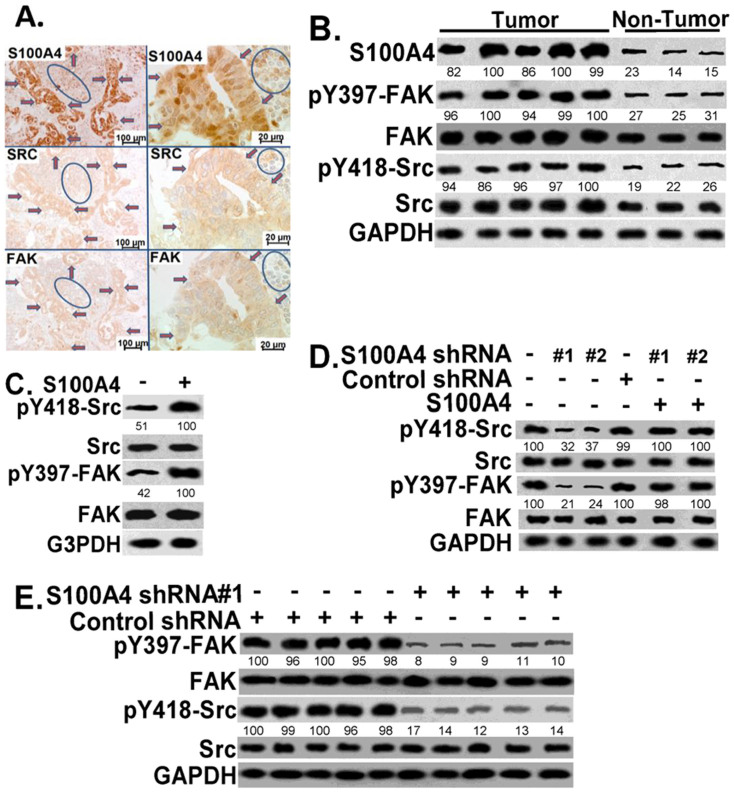
S100A4 promotes Src and FAK activation; S100A4-deficient pancreatic tumors show reduced Src and FAK activation in pancreatic cancer animal model. (A) This figure demonstrates the phenotypic expression of S100A4, SRC, and FAK in the same area of a metastasis of pancreatic cancer to a lymph node. A low power photograph (original magnification ×100) and the higher power photograph (original magnification ×630) are shown. Malignant cells are indicated by red arrows with blue outlines. Areas of lymphocytes are within the blue circles. (B) Human pancreatic tumor tissues (n = 5) and control non-tumor pancreatic tissues (n = 3) were lysed, and equivalent amounts of whole tissue lysates were Western blotted with indicated antibodies. Membranes were probed with antibodies for phospho-FAK-Tyr397 (pY397-FAK) and phospho-Src-Tyr418 (pY418-Src) for FAK and Src activation, respectively. (C) S2VP10 cells were serum starved and followed by vehicle or S100A4 (1 μg/ml) treatment in serum free medium for 6 hours. Cells were lysed, and equivalent amounts of whole cell lysates were Western blotted with indicated antibodies. (D) S2VP10 cells infected with S100A4 or control shRNAs were lysed, and treated with or without S100A4 (1 μg/ml) as panel (C), and equivalent amounts of whole cell lysates were Western blotted with indicated antibodies. (E) FAK and Src activation *in vivo* in S100A4-deficient and control tumors were examined. Pancreatic tumors were obtained from mice as shown in Figure 3, lysed, and equivalent amounts of whole tissue lysates were Western blotted with indicated antibodies. GAPDH was used as loading control. Each lane represents one individual tumor sample. The experiments were repeated 3–4 times and representative images are shown.

Aberrant S100A4 expression, and increased FAK and Src activation, have been linked to pancreatic cancer progression^7^,^13^,^19^,^20^,^31^. To understand whether S100A4 is coordinating with FAK and Src to promote pancreatic cancer, phenotypic expression of these molecules was examined in human pancreatic tumors and non-tumor control tissues. Immunohistochemical studies showed the co-expression of S100A4, FAK and Src protein in the same area of a lymph node metastasis from a human primary pancreatic adenocarcinoma sample (Fig. 4A). The levels of FAK activation (pY397-FAK) and Src activation (pY418-Src) were significantly increased in primary human pancreatic adenocarcinoma when compared to that in control (non-tumor) tissues (Fig. 4B). These results suggest a potential association of S100A4 with FAK and Src in pancreatic cancer. To further understand the role of S100A4 in Src and FAK signaling, pancreatic cancer cells were treated without or with S100A4 (1 μg/ml) in serum free medium for 6 hours. Enhanced Src and FAK activation were observed in S2VP10 cells in response to S100A4 treatment (Fig. 4C). In contrast, S100A4 downregulation resulted in decreased Src and FAK activation in S2VP10 cells (Fig. 4D). The effect of S100A4 downregulation on Src and FAK activation can be rescued by S100A4 treatment (Fig. 4D), supporting that the effect of S100A4 downregulation on Src and FAK is specific. Animals injected with S100A4-deficient S2VP10 cells showed a significant delay in tumor development (Figs. 3A and 3B above), and these S100A4-deficient tumors had remarkably reduced FAK and Src activation (when compared to control tumors) (Fig. 4E and Supplementary Fig. S2C). These findings indicate that S100A4 is critical in promoting Src and FAK activation in pancreatic cancer cells and tumors.

### Src and FAK are partially dependent upon each other, and they form a dual signaling pathway that regulates pancreatic cancer cell progression

**Figure 5:**
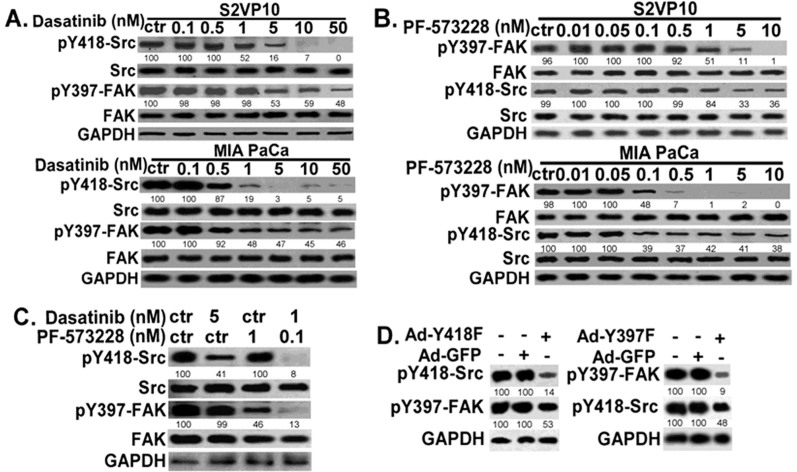
Src and FAK activation partially depends on each other in pancreatic cancer cell lines. S2VP10 and MIA PaCa-2 (MIA PaCa) cells were treated with (A) Src inhibitor (Dasatinib) or (B) FAK inhibitor (PF-573228). Control (ctr) cells from same lines were treated with vehicle only. Cells were lysed and equivalent amounts of whole tissue lysates were Western blotted with indicated antibodies. (C) S2VP10 cells were treated without or with Dasatinib, PF-573228, or both at indicated doses, and cell lysates were subjected for Western blot analysis with indicated antibodies. (D) Overexpression of dominant negative Src mutant (Y418F-Src) was mediated by an adenoviral vector (shown in left panel). Representative Western blot image of overexpression of dominant negative FAK mutant (Y397F-FAK) mediated by an adenoviral vector is shown in right panel. Cells were lysed, and equivalent amounts of whole tissue lysates were Western blotted with indicated antibodies for Src and FAK activation. The experiments were repeated 3–4 times and representative images are shown. GAPDH was used as loading control.

The above data demonstrate that S100A4 promotes pancreatic cancer cell progression *in vitro* and *in vivo*, and the effects of S100A4 are likely at least mediated by downstream signaling of FAK and Src. Activation of FAK and Src has been reported to be dependent upon each other; however these studies were performed mostly in non-tumor cells^22^. The interaction of Src and FAK has not been rigorously tested in aggressive pancreatic cancer cells, and their inter-dependence could be different in malignant cells. To characterize their interaction in the context of pancreatic cancer, S2VP10 and MIA PaCa-2 cells were treated with Src inhibitor (Dasatinib) or/and FAK inhibitor (PF573228), followed by Western blot analysis for Src and FAK activation (Figs. 5A and 5B). As expected, Dasatinib inhibited Src activation in a dose dependent manner; however, the effect of Dasatinib on FAK activation was not totally dose dependent (Fig. 5A). FAK activation was only partially blocked by Dasatinib (ranging from 40–60%) while Src activation was completely blocked at the same dose (such as 10 nM) (Fig. 5A), suggesting that in pancreatic cancer cells FAK activation is not totally dependent upon Src. Similarly, PF573228 inhibited FAK activation in a dose dependent manner; however, Src activation was only partially blocked while FAK activation is completely blocked at the same dose (10 nM) (Fig. 5B). These findings support that FAK and Src signaling in pancreatic cancer cells are not completely dependent upon each other.

**Figure 6:**
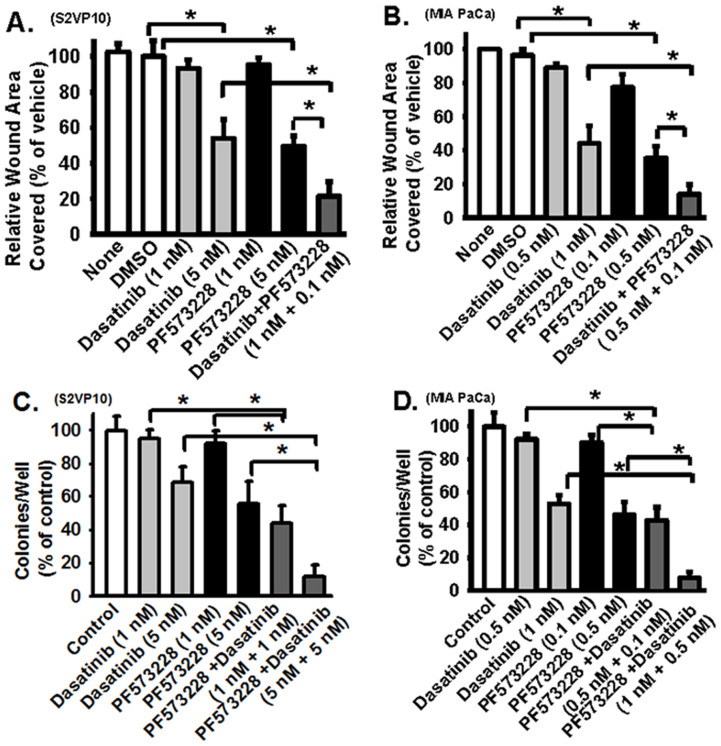
Pancreatic cancer migration and growth are regulated by the dual signaling pathway mediated by both Src and FAK. S2VP10 cells or MIA PaCa cells were treated with vehicle (none or DMSO) or with Dasatinib, PF-573228, or both Dasatinib and PF-573228. Cells were analyzed in wound closure cell motility assays (24 hours, shown in panels A and B) or soft agar growth assays (14 days) (shown in panels C and D). The experiments were repeated 3–4 times with 2–3 replicates. Data are represented as mean + SE. * represents p < 0.01 for the indicated two groups.

Expression of a Src dominant negative mutant (Y418F-Src) also did not completely inhibit FAK activation (Fig. 5D, left panels). Similarly, expression of a dominant negative FAK mutant (Y397F-FAK) did not completely inhibit Src activation (Fig. 5D, right panels), again supporting that FAK and Src are only partially dependent upon each other. When Dasatinib and PF573228 were used together, both Src and FAK activation can be effectively blocked at a relatively lower dose (relative to single inhibitor) (Fig. 5C), indicating that FAK and Src do reciprocally activate each other in pancreatic cancer cell lines. Inhibition of both Src and FAK activation by inhibitors induces cleavage of caspase-3 and PARP and increased cell apoptosis in pancreatic cancer cells (supplementary Fig. S6). Cell migration and growth were inhibited by Dasatinib or PF573228 in a dose dependent manner (Figs. 6A–6D). Compared to Dasatinib or PF573228 used alone, when they were used together, the same dose can achieve better inhibitory effect or a relatively lower dose can achieve similar inhibitory effect on cell migration and growth (Figs. 6A–6D), suggesting that it may be beneficial to use FAK and Src inhibitor together in pancreatic cancers.

## Discussion

This study demonstrates the critical role of S100A4 in tumorigenic potential of pancreatic cancer cells, such as migration, invasion, growth, and survival. S100A4 downregulation in tumor cells greatly impairs their ability to develop tumors *in vivo*. S100A4 expression is necessary to protect pancreatic cancer cells against TGFβ-induced growth inhibition and apoptosis. S100A4 promotes activation of Src and FAK, which form a dual signaling pathway and contribute to the tumorigenic capacity of pancreatic cancer cells.

A large number of reports demonstrate that increased S100A4 is significantly correlated with tumor invasion and metastasis^3^,^15^. Western blot analysis of human pancreatic tissues revealed a high level of S100A4 expression (Supplementary Fig. S1). Our results are consistent with previous reports that S100A4 is overexpressed in pancreatic cancer^7^. Increased S100A4 expression is an independent biomarker for poor outcomes of pancreatic cancer^7^,^13^; however, its implications in pancreatic cancer progression remain to be adequately tested. Animals injected with S100A4-deficient tumor cells have significantly smaller pancreatic tumors relative to controls (Fig. 3), strongly supporting a direct role of S100A4 in pancreatic cancer progression *in vivo*. S100A4 likely promotes pancreatic cancer progression *in vivo* through multiple mechanisms. *In vitro* S100A4 plays an important role in pancreatic cancer cell lines in cell migration and invasion, anchorage-independent growth, proliferation, and survival (Figs. 1 and 2). Importantly, S100A4-deficient tumors show decreased proliferation signaling (increased p27^Kip1^ and decreased cyclin E) and increased apoptotic signaling (cleaved caspase-3 and PARP) (Fig. 3 and Supplementary Fig. S2). Our *in vitro* findings are consistent with previous *in vitro* studies, that reported S100A4 regulates tumor cell invasion, proliferation, angiogenesis in thyroid cancer cells^42^ or regulates invasion in human pancreatic cancer cells^43^. The *in vivo* and *in vitro* data are consistent and support the effects of loss of S100A4 in inhibition of tumor progression. One limitation is that the effects of gain of S100A4 *in vivo* in tumor progression have not been specifically tested, and future studies are necessary to understand the effects of gain of S100A4 in tumor progression *in vivo*. Nonetheless, this work also advances our knowledge by providing evidence demonstrating that S100A4 plays an important and direct role in pancreatic tumor growth *in vivo*.

Interestingly, while TGF-β1 has no effect on control pancreatic tumor cells, TGF-β1 is able to induce significant growth inhibition and apoptosis in S100A4-deficent pancreatic tumor cells (Fig. 2). The findings indicate that S100A4 is involved in mechanisms by which pancreatic cancer cells escape from TGFβ-induced effects (such as anti-proliferation). This is important as there is abundant TGFβ present in the tumor microenvironment. Reversing the insensitivity of pancreatic tumor cells to TGFβ would be critical to limiting the progression of pancreatic tumors. Our data support that S100A4 has a non-canonical function in pancreatic cancer progression. Whether S100A4 directly or indirectly regulates the TGFβ resistant phenotype, and the identification of the mechanisms involved, are beyond the scope of the current study and will be addressed in the future. Some evidence suggests that S100A4 may regulate tumor progression through modulating the tumor environment. VEGFA expression is decreased in S100A4-deficient tumors (Fig. 3). This is consistent with a recent report showing that downregulation of S100A4 decreases VEGF expression in thyroid cancer cells^42^. Also, others reported a high incidence of hemangiomas–benign tumors of vascular origin, in transgenic mice ubiquitously expressing S100A4^44^, and S100A4-mediated endothelial cell motility might be another mechanism for the increased angiogenesis seen in these animals. Based on these findings, we speculate that S100A4 stimulates pancreatic cancer progression directly by promoting tumorigenic potential of cancer cells and indirectly by modulating tumor microenvironment including angiogenesis. How S100A4 regulates VEGFA expression in tumor cells, and whether it is directly or indirectly regulated, are interesting questions that remain to be answered. A recent study further supports the role of S100A4 in pancreatic cancer progression by promoting angiogenesis^45^. The study demonstrates that S100A4 supports tumorigenesis likely through synergizing with VEGF to promote endothelial cell migration and by increasing MMP-9 activity. Inhibition of S100A4 signaling by S100A4 shRNA or a neutralizing monoclonal antibody against S100A4 dramatically decreases tumor development of the pancreatic MiaPACA-2 cell line injected subcutaneously in nude mice^45^. Our findings are consistent with their findings, that targeting S100A4 or its critical pathways is an attractive approach for the treatment of human pancreatic cancer.

Src and FAK are frequently upregulated in pancreatic tumors^19^,^20^,^23^,^31^. Similar to S100A4, increased Src or FAK signaling is associated with a poor prognosis of pancreatic cancer^19^,^20^,^23^,^31^. However, little is known about the interaction among S100A4, Src, and FAK in pancreatic cancer cells. Our findings indicate that the activity of S100A4 is mediated at least by Src and FAK signaling pathways. S100A4 enhances Src and FAK activation; in contrast, S100A4 downregulation clearly reduces Src and FAK activation (Fig. 4). Increased S100A4 expression is associated with increased Src and FAK activation in human pancreatic tumor tissues, and S100A4-deficient tumors show remarkably decreased FAK and Src activation *in vivo* in a pancreatic cancer mouse model (Fig. 4E and Supplementary Fig. S2). These findings support that S100A4 plays an important role in modulating Src and FAK activation. We have previously shown that FAK regulates brain tumor cellular proliferation through cyclin D1 and p27^Kip1^^34^. This study provides evidence that S100A4 regulates cyclin E and p27^Kip1^ expression *in vitro* and *in vivo* in pancreatic tumor cells. We speculate that S100A4 regulates cyclin E and p27^Kip1^ expression at least through FAK-mediated signaling, and this will be addressed by future studies.

In other systems, such as fibroblasts, a cross-talk between Src and FAK is necessary for their activation and maximal activation^22^. The SH2 domain of Src binds to the tyrosine 397 (Y397) of FAK, leading to phosphorylation of multiple sites of FAK, resulting in maximal FAK activation^22^,^32^,^46^. Meanwhile, FAK is important for Src activation. FAK binds to the SH2 domain of Src, leads to the release of Src kinase domain from auto-inhibition (by Y531 of Src), and results in Src activation^29^,^32^,^47^. Inhibition of Src (or FAK) usually results in inhibition of FAK or Src)^21^,^22^,^32^. However, our results argue that although S100A4 stimulates both Src and FAK signaling, activation of FAK and Src are not totally dependent upon each other in pancreatic cancer cells. This is demonstrated by the finding that the complete inhibition of Src (or FAK) did not completely inhibit FAK (or Src) activation (Fig. 5). Only when both Src and FAK inhibitors were used at the same time, was the activation of Src and FAK blocked (Fig. 5). Furthermore, overexpression of dominant negative Y418F-Src mutant (or Y397F-FAK mutant) did not completely inhibit FAK (or Src) activation, again supporting that (1) Src and FAK are not totally dependent upon each other, and (2) they form a dual signaling pathway in pancreatic cancer cells.

Although they are not totally dependent upon each other, FAK and Src reciprocally activate each other in pancreatic cancer cells. Blocking Src (or FAK) activation reduces FAK (or Src) activation (Fig. 5). This is important as Src and FAK form a reciprocal activation mechanism or a feed-forward loop that greatly enhances both Src and FAK activity and the aggressive aspects of pancreatic tumor cell lines. There are ongoing clinical trials using Src or FAK inhibitors to treat various cancers (including pancreatic cancers, clinicaltrials.gov). Previous studies show that Src inhibitor (Dasatinib) reduces pancreatic tumor mass in an animal model, but the resulting tumor mass remained large^31^. One recent phase I/II study reports that Src inhibitor (AZD0530) in combination with gemcitabine did not improve efficacy over what would be expected from gemcitabine alone in advanced pancreatic cancer^48^, suggesting that Src inhibition alone might not be beneficial. Because evidence suggests that both Src and FAK mediate the aggressive behavior of pancreatic cancer cells and that they form a dual signaling pathway (Figs. 5 and 6), it might be beneficial to study whether a combination of Src and FAK inhibitors, with or without other current therapeutic agents, will enhance their overall efficacy to limit pancreatic cancer progression *in vivo*.

In summary, the current work supports the role of S100A4 in pancreatic cancer progression *in vivo*, and provides novel insights into the signaling mechanisms by which S100A4 promotes tumorigenic capacity of pancreatic carcinoma cells. S100A4 expression is important for pancreatic tumor cells to avoid TGF-β1-induced growth inhibition and apoptosis. S100A4 enables FAK- and Src-mediated signaling that operate as a dual signaling pathway and underlie the tumorigenic potential of pancreatic carcinoma cells. These findings suggest alternative strategies to the current therapeutic approaches targeting FAK and Src. The inhibition could be achieved by disabling the S100A4 function or by inactivating Src and FAK pathways. These approaches may be advantageous for treatment of aggressive or advanced pancreatic cancer, which are not manageable by most of the current therapies.

## Methods

### Antibodies and other reagents

The following antibodies were purchased: S100A4 (Dako, Carpinteria, CA); phospho-FAK [pY397-FAK] (Biosource, Camarillo, CA); p27Kip1, cyclin E, Src, and FAK (Santa Cruz, CA); phospho-SrcY418, cleaved caspase-3, cleaved Poly-(ADP-ribose) polymerase (PARP), (EMD Millipore, Billerica, MA); and glyceraldehyde 3-phosphate dehydrogenase (GAPDH) (Research Diagnostics, Flanders, NJ). TGF-β1 and S100A4 were purchased from R&D Systems (Minneapolis, MN, USA). Dasatinib (Src kinase inhibitor) was purchased from LC Laboratories (Woburn, MA). PF573228 (FAK inhibitor) was purchased from Calbiochem (Calbiochem, Millipore, Billerica, MA). Chemicals were purchased from Sigma-Aldrich (St. Louis, MO) and Fisher Scientific (Waltham, MA). TGF-β1 and S100A4 were purchased from R&D Systems (Minneapolis, MN, USA). The pan-caspase inhibitor Z-VAD-fmk was purchased from BD PharMingen (San Diego, CA). Chemicals were purchased from Sigma-Aldrich (St. Louis, MO) and Fisher Scientific (Waltham, MA).

### Human pancreatic cells and cell culture

Propagation and maintenance of MIA PaCa-2 and S2-VP10 cells were described previously^49^. MIA PaCa-2, Panc 2.03, BXPC3 cells were obtained from the American Type Culture Collection. S2-VP10 cells were described previously and provided by Dr. Michael A. Hollingsworth at the University of Nebraska (Omaha, NE)^50^. Cells were grown in Dulbecco's-modified Eagle's medium containing 4.5 g/L glucose (DMEM, Mediatech Inc.) supplemented with 10% fetal bovine serum (FBS, Hyclone), 2 mM L-glutamine, and antibiotics^49^. S2VP10 and MIA PaCa-2 cells were treated with or without S100A4 (1 μg/ml) and subjected to indicated assays. For transforming growth factor beta-1 (TGF-β1) treatment, S2VP10 and MIA PaCa-2 cells were serum starved with serum-free medium (DMEM with 1% BSA) for 18–24 hours, followed by TGF-β1 (4 ng/ml) treatment for the indicated time in serum-free medium. For caspase inhibitor assays, the above cells were also treated with or without pan-caspase inhibitor Z-VAD-fmk (20 μmol/L) or vehicle for 24 hours and subjected to Annexin V apoptosis assay.

### Human pancreatic tumor and non-tumor control tissues

The studies and protocols have obtained approvals from local IRB. All experiments were performed in accordance with relevant guidelines and regulations. De-identified tumor tissues (n = 5), and non-tumor control tissues including matched adjacent non-tumor tissues (n = 2) and non-tumor tissue (n = 1), were obtained through UAB tissue procurement program.

### Human pancreatic cancer orthotopic xenograft mouse model

All animal interventions were approved by local IACUC. All experiments were performed in accordance with relevant guidelines and regulations. The model was performed as described by us previously^40^. Briefly, female severe combined immunodeficient (SCID) mice at 2 months of age (Harland Laboratory) were allowed to acclimate for 4 weeks before use. Mice were anesthetized, followed by orthotopic cell implantation. Pancreatic cancer cell suspension was stored on ice in a sterile tube and then drawn up using a 28-gauge needle to aliquot 1 × 10^5^ cells/30 μl which was injected into the tail of the pancreas after the following procedures. A sterile cotton tipped applicator was used to cover the injection site for 30s to prevent peritoneal leakage. The organs were returned to the abdomen and the skin and peritoneum were closed in a single layer closure with 5-0 Prolene sutures. Animals recovered on a warming blanket and received liquid acetaminophen for 24 hours with food and water ad libitum. Animals were euthanized at day 21 post xenograft procedure. Primary tumors in the pancreas were excised and the final tumor volume was calculated by using the modified ellipsoidal formula: tumor volume = 1/2 × L × W × H (where L = length, W = width, and H = depth or high). The L, W, and H were determined by using external caliper. The ellipsoid volume formulas (1/2 × L × W × H) is one of the best approaches for estimating tumor mass in animal models^51^,^52^.

### Lentiviral vectors for S100A4 downregulation and stable clones

The replication incompetent lentiviral vectors expressing short hairpin RNA (shRNA) for silencing S100A4 (two shRNAs) and lentiviral vectors expressing control non-targeting shRNA were from OpenBioSystem Thermo Scientific (Pittsburgh, PA). Pancreatic cancer cells were transfected with lentiviral vectors according to manufacturer's instructions and as described previously^53^. Stable pancreatic cancer cell clones expressing the above shRNAs were selected and maintained in medium containing 9 μg/ml puromycin according to the manufacturer's instructions, and screened by Western blot analysis (for protein level) and quantitative RT-PCR (for mRNA level) as described by us previously^54^.

### Preparation, purification, and infection of the adenoviral vectors (Ad-Y397F and Ad-Y418F) and control adenoviral-GFP (Ad-GFP) constructs

Ad-Y397F is an adenoviral vector containing the hemaglutinin (HA)-tagged phospho-FAK mutant cDNA (Y397F-FAK, dominant negative mutant). Y397F-FAK is where tyrosine 397 of FAK is replaced by phenylalanine (Y397F is unable to be phosphorylated/activated). The construction of the adenoviral vector used similar procedures as described previously^55^. Ad-Y418F is an adenoviral vector containing hemaglutinin (HA)-tagged phospho-Src mutant cDNA, Y418F-Src, a dominant negative mutant where tyrosine 418 of Src is replaced by phenylalanine. The preparation, purification, and infection of adenoviral vectors, and the green fluorescent protein (GFP) expression mediated through the same adenoviral vector (Ad-GFP) were described previously^55^.

### Western blot analysis

Human pancreatic tumor cells or pancreatic tumor tissues were lysed in RIPA lysis buffer with 1% deoxycholate, 1.0% Triton X-100, and 0.1% SDS in 0.01 M Tris base (pH 7.4), 0.15 M NaCl, and containing the following inhibitors, 100 μM phenylmethanesulfonyl fluoride (PMSF), 10 μg/ml aprotinin, 10 μg/ml leupeptin, and 100 μM sodium vanadate, essentially as described previously^53^. Protein concentration of whole cell or tissue lysate was determined by BCA kit (Pierce, Rockford, IL). Equivalent micrograms of whole cell or tissue lysates were electrophoresed on SDS PAGE, transferred to Immobilon-P membrane (Millipore Corp., Bedford, MA), probed with indicated antibodies, and developed with ECL system (Pharmacia Biotech, Piscataway, NJ) as described^56^. Densitometry analysis of band density was described previously^53^. The densitometric readings were pooled and averaged from three independent experiments. Density was relative to GAPDH and the relative highest band density was as 100%. The background of densitometric reading on the ECL-developed film was subtracted. The expression of GAPDH protein was used as a loading control.

### Cell migration and invasion assay

The wound closure monolayer/scratch motility assay and invasion assay were performed as described previously^55^. Briefly, cells were plated in serum-free DMEM with 1% BSA for 24 hours. Mitomycin C was added to inhibit cell proliferation. The monolayer was scratched, and the wound area covered by cell migration over 24 hours on digital photomicrograph images was calculated. *In vitro* invasion assay was performed with the kit with matrigel-coated inserts according to the manufacturer's instructions (BD Biosciences, San Jose, CA). 1 × 10^5^ cells/well was added to the upper compartments of the invasion chamber. The values obtained were calculated using the number of invaded cells from three filters after 24 hours. Final results were pooled from two to three individual experiments.

### Quantitative Real-Time RT-PCR Analysis

Quantitative RT-PCR was performed as described by us previously^54^,^56^. Briefly, total RNA is extracted from pancreatic tumor cells or tissues using RNeasy Mini Kit (Qiagen, Valencia, CA) according to the manufacturer's instructions. All samples were treated by DNAse according to the manufacturer's instructions. The following primers were used: for S100A4, sense 5′- GGTGTCCACCTTCCACAAGT-3′ and antisense 5′-GCTGTCCAAGTTGCTCATCA-3′; for vascular endothelial growth factor (VEGFA), sense 5′-GAGCAAGACAAGAAAATCCC-3′ and anti-sense 5′- CCTCGGCTTGTCACATCTG-3′; and for glyceraldehyde 3-phosphate dehydrogenase (GAPDH) sense 5′-GAGTCAACGGATTTGGTCGT-3′ and antisense 5′-TTGATTTTGGAGGGATCTCG-3′. One to three microgram of total RNA was reverse transcribed to cDNA with Maloney murine leukemia virus reverse transcriptase (Promega, Madison, WI). Quantitative RT-PCR analysis was carried out with the SYBR® Green PCR Master Mix (Applied Biosystems, Foster City, CA) using the Roche Light Cycler 480 (Indianapolis, IN). Samples were assayed in triplicate, and the values were normalized to the relative amounts of GAPDH.

### Annexin V apoptosis assay

The Annexin V assay with propidium iodide labeling was performed with apoptosis kit (BD Pharmingen, San Diego) on pancreatic tumor cells with indicated treatment as per the manufacturer's instructions and described previously by us^34^.

### Cell proliferation assay

Cell proliferation assays were done in 6-well plates in serum-free medium (DMEM with 1% BSA) as previously described^34^. A total of 2.5 × 10^5^ cells were plated per well, treated with vehicle or TGF-β1 (4 ng/ml), and the total number of cells per well were counted after 24 hours in five replicates per condition. Experiments were repeated at least twice.

### Soft agar growth assays

Anchorage-independent growth assays were performed as described previously^34^. Briefly, pancreatic tumor cells were harvested and resuspended in a 0.3% agar, poured onto plates containing a layer of 0.5% agar, and then maintained at 37°C, 5% CO2 for 14 days. The resulting colonies were counted with a colony being defined as >10 cells. Samples were assayed in replicates of three, and the experiment was repeated three times.

### Immunohistochemistry

Analysis of S100A4, FAK, and Src on human pancreatic tumor tissue sections were performed as described previously^34^. Briefly, paraffin tissue sections were cut at 4 μm, and paraffin was removed by using xylene and denatured alcohol, incubated with indicated primary antibody (4°C) overnight, then primary antibodies were detected with a HRP-linked secondary antibody (Jackson ImmunoResearch, West Grove, PA, 1 hr at RT), and developed using a 3,3'-diaminobenzidine (DAB) substrate kit. Primary antibody was deleted in the negative control (data not shown).

### Cell Cycle Analysis

Cell cycle analysis by flow cytometry was performed as described previously by us^34^. Briefly, cells were harvested with trypsin, washed with cold phosphate-buffered saline, fixed in 70% ethanol, and kept at 4°C overnight. Fixed cells were washed with phosphate-buffered saline and resuspended in 40 microgram/ml RNase A and 40 microgram/ml propidium iodide in phosphate-buffered saline, then analyzed using a FACScan for DNA content (BD Biosciences, FACS Calibur).

### Vascular Endothelial Growth Factor (VEGF) ELISA

The VEGF ELISA was performed as per the instructions with the Quantikine Human VEGF Immunoassay ELISA Kit (R&D Systems, Minneapolis, MN, USA). Similar ELISA procedures were described by us previously^57^.

### Statistical Analysis

Data were analyzed using the student t-test analysis (Sigma Plot, SPSS Inc.) for differences between two groups, and findings were expressed as mean + SE. Experiments were repeated three to four times. A p value < 0.05 was considered to be statistically significant.

## Author Contributions

P.C., W.G. and Q.D. wrote the main manuscript text and P.C., Y.Y., J.S., A.L., M.H. and G.-Q.C. largely contributed to the animal studies. X.H., D.J.B., J.D.C., Q.T., D.C., Q.L. and Y.Y.L. largely contributed to cell signaling studies in cells and tissues. All authors contributed to the figures and supplemental figures and reviewed the manuscript.

## Supplementary Material

Supplementary Information Supplementary Figure and Legend
