# Supplementary material for: S100A4 promotes pancreatic cancer progression through a dual signaling pathway mediated by Src and focal adhesion kinase
Source: Sci Rep. 2015 Feb 13;5:8453. doi: 10.1038/srep08453 (PMC4326725; doi:10.1038/srep08453)
Supplement: Supplementary Information — Supplementary Figure and Legend [file srep08453-s1.pdf]

S100A4 promotes pancreatic cancer progression through a dual signaling pathway mediated by Src and focal adhesion kinase

Pulin Che, Youfeng Yang, Xiaosi Han, Meng Hu, Jeffery C. Sellers, Angelina I. Londono-Joshi, Guo-Qiang Cai, Donald J Buchsbaum, John D Christein, Qinjiu Tang, Dongquan Chen, Qianjun Li, William E Grizzle, Yin Ying Lu, and Qiang Ding

Supplementary Figure S1

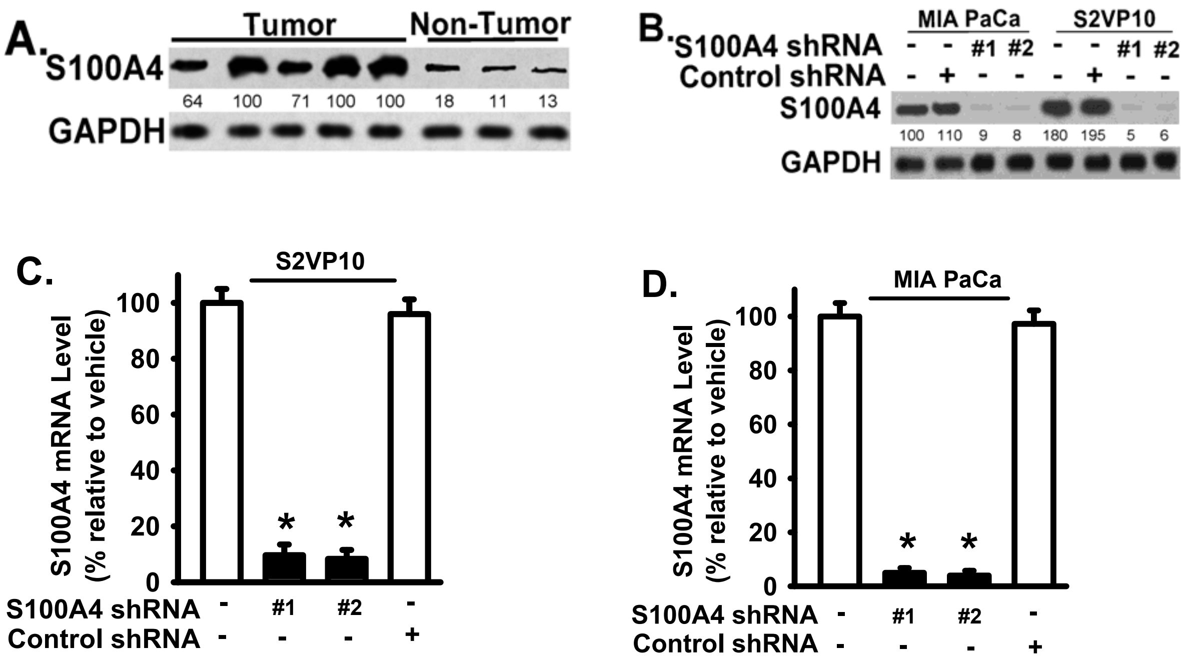

Supplementary Figure S2

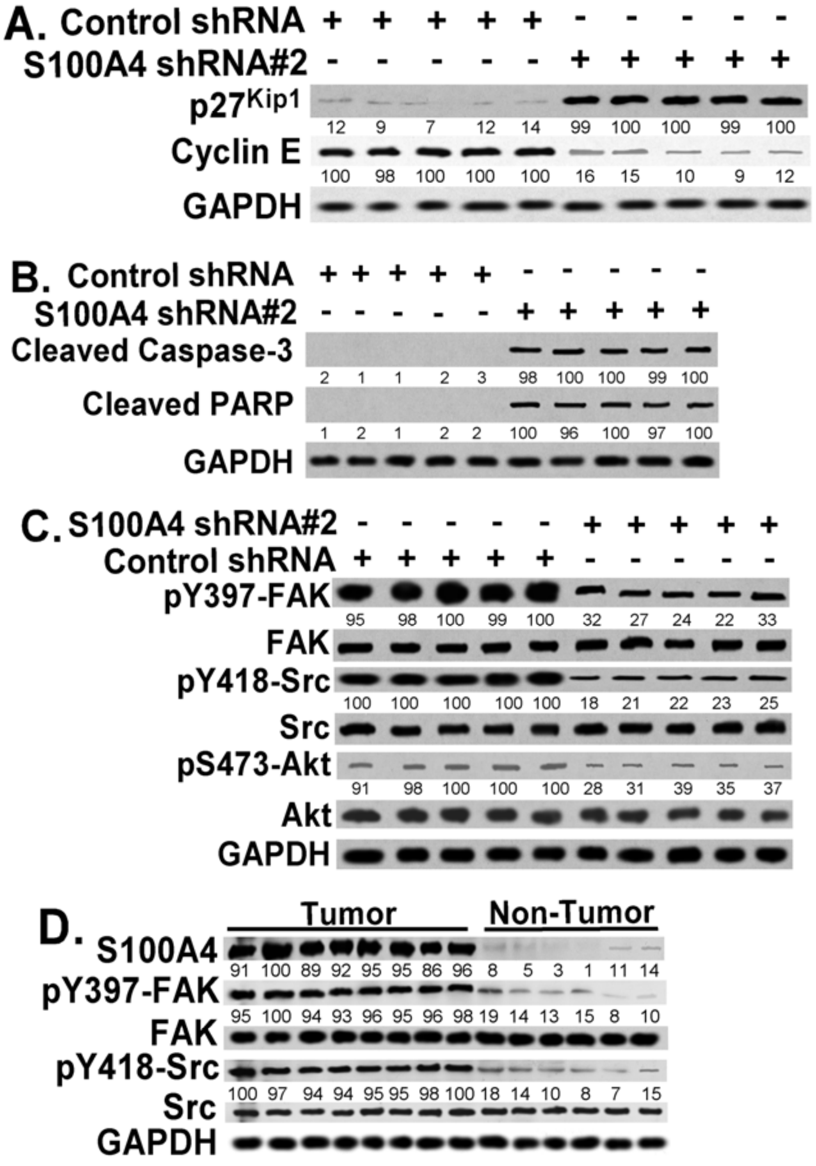

Supplementary Figure S3

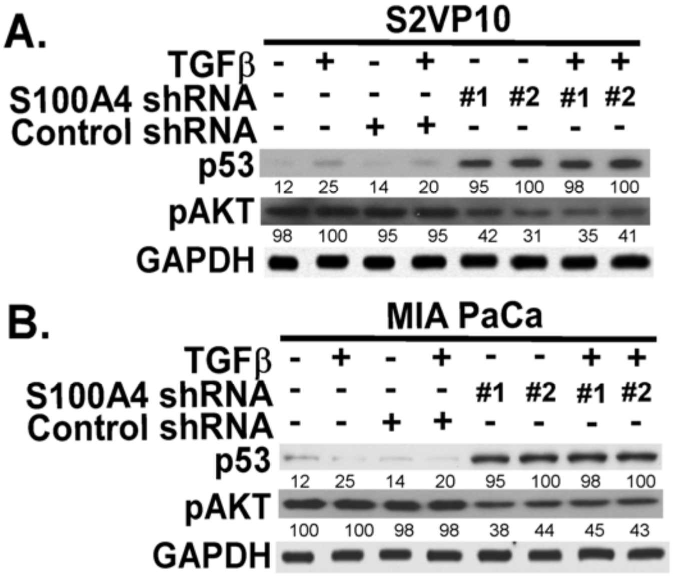

Supplementary Figure S4

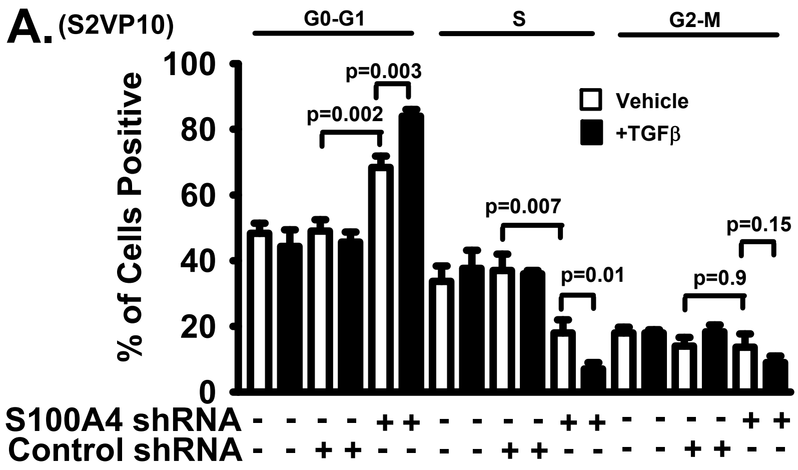

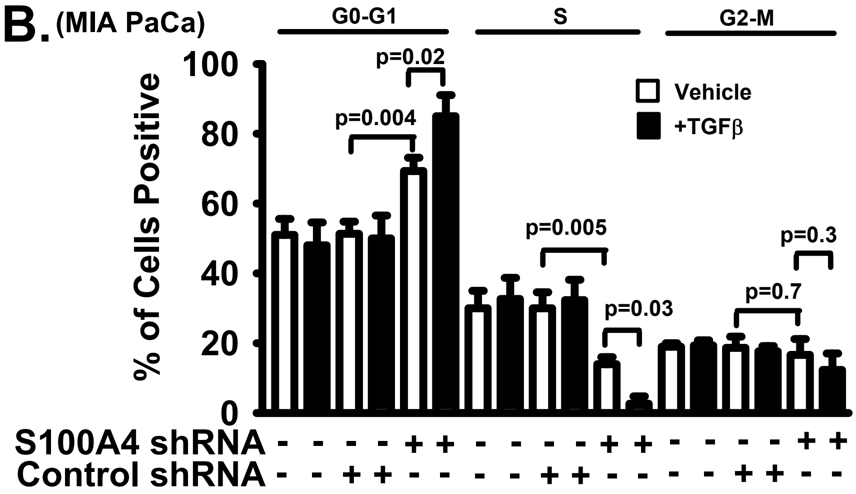

Supplementary Figure S5

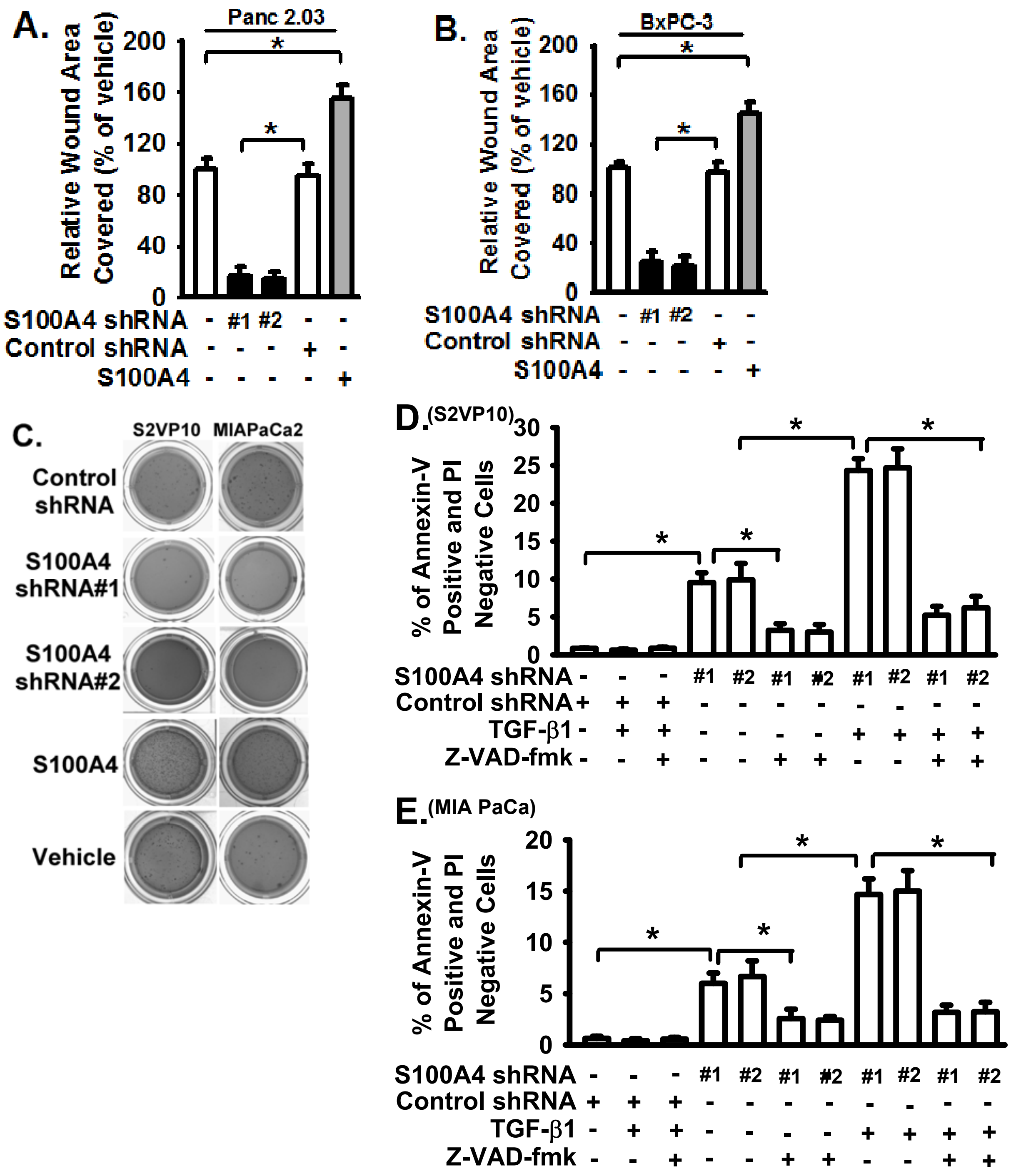

Supplementary Figure S6

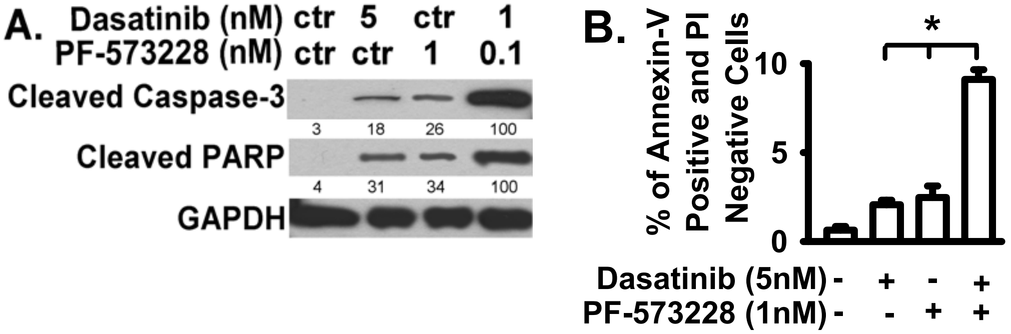

Supplementary Figure 7

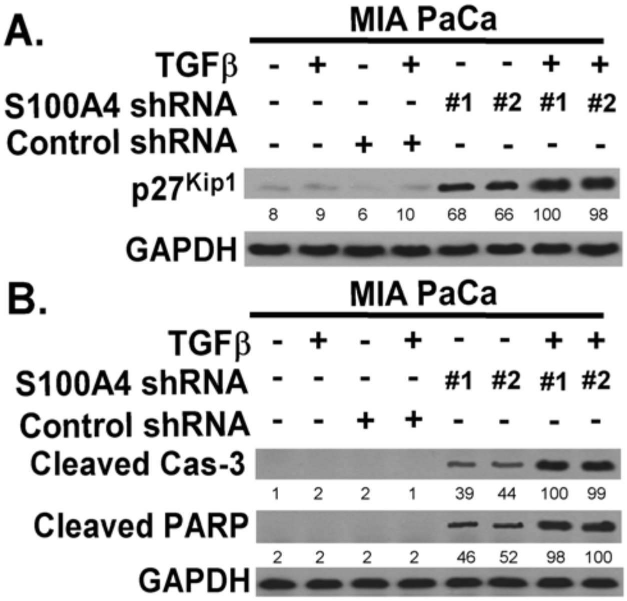

Supplementary Figure Legend

Supplementary Figure S1.

S100A4 expression is increased in human pancreatic tumors; S100A4 downregulation in pancreatic cancer cell lines by short hairpin RNA (shRNA)

(A) Human pancreatic tumor tissues (n = 5) and control non-tumor pancreatic tissues (n = 3) were lysed, and equivalent amounts of whole tissue lysates were Western blotted with anti-S100A4 antibody. GAPDH was used as a loading control. (B) MIA PaCa-2 (labeled as MIA PaCa in all figures) and S2VP10 pancreatic cancer cells were infected with lentiviral vectors containing S100A4 shRNA #1, S100A4 shRNA #2, or control non-targeting shRNA, and stable clones were selected according to puromycin resistance as described in Materials and Methods. Cells were harvested and lysed. Equivalent amounts of whole cell detergent lysates were Western blotted with the indicated antibodies. (C - D) S2VP10 cells in panel C and MIA PaCa-2 (MIA PaCa) cells in panel D, stably expressing the above S100A4 shRNA #1, #2, or non-targeting control shRNA were subjected to total RNA extraction, and followed by quantitative RT-PCR to determine the level of S100A4 mRNA. The experiments were repeated 3-4 times. Data are represented as mean + SE. * represents $p < 0.001$ for cells with S100A4 shRNA compared with cells with control shRNA.

Supplementary Figure S2.

S100A4 downregulation significantly reduces expression of cyclin E, inhibits activation of FAK and Src, and increases p27^{Kip1} expression and levels of cleaved caspase-3 and PARP *in vivo* in pancreatic cancer mouse model

(A – C) S2VP10 cells stably infected with S100A4 shRNA #2 or control shRNA were used in a human pancreatic cancer orthotopic xenograft mouse model. Primary tumors in the pancreas were excised at day 21 post injection. Tumor tissues were lysed and equivalent amount of whole tissue lysates were Western blotted with indicated antibodies. (D) Human pancreatic tumor tissues (n = 8) and control non-tumor pancreatic tissues (n = 6) were lysed, and equivalent amounts of whole tissue lysates were Western blotted with indicated antibodies. The experiments were repeated 3-4 times and representative images are shown. GAPDH was used as loading control.

Supplementary Figure S3.

S100A4 downregulation increases P53 expression and decreases Akt phosphorylation, and TGF-β1 fails to enhance the effect of S100A4 downregulation on P53 expression and decreases Akt phosphorylation in pancreatic tumor cell lines

S100A4 downregulation was achieved with lentiviral vectors as shown in Fig. 1. S2VP10 and MIA PaCa-2 (MIA PaCa) cells with or without shRNA infection were serum starved with serum-free medium (DMEM with 1% BSA) for 24 hours, followed by treatment with TGF-β1 (4 ng/ml) or vehicle for 24 hours in serum-free medium. Cells were lysed, and equivalent amount of lysates were Western blotted with indicated antibodies. Phosphorylation within the carboxy terminus of Akt at Ser473 was examined (pAkt). The experiments were repeated 3-4 times and representative images are shown. GAPDH was used as loading control.

Supplementary Figure S4.

S100A4 downregulation inhibits cell cycle progression and TGF-β1 sensitizes the effect of S100A4 downregulation on cell cycle progression in pancreatic tumor cell lines

S100A4 downregulation in S2VP10 (Panel A) and MIA PaCa-2 (MIA PaCa in Panel B) cells was achieved with lentiviral vectors, then cells were treated with TGF-β1 (4 ng/ml) or vehicle as supplementary Fig. S3, and followed by

propidium iodide labeling and FACS analysis for DNA content. Note that the percentage of cells in G0/G1 (shown by bars 1 to 6), S (shown by bars 7 to 12), and G2/M (shown by bars 13 to 18) phases is plotted as a histogram, and the statistical significance of differences in each phase when cells were TGF-β1 treated (black bars) versus vehicle treated (blank bars) is indicated by the p value; $p < 0.05$ was considered significant. The experiments were repeated 3 times. Data are represented as mean + SE.

Supplementary Figure S5.

S100A4-downregulation reduces cell migration and anchorage-independent growth, and induces caspase-mediated apoptosis in pancreatic tumor cell lines

S100A4-downregulation was achieved with lentiviral vectors as shown in supplementary Figure S1. Cells were also treated with or without S100A4 (1 μg/ml), before subjected to the wound closure motility and anchorage-independent/soft-agar growth assays as shown in Figure 1. (A – B) Wound closure motility assays in Panc 2.03 and in BXPC3 pancreatic cancer cell lines. (C) Representative images of anchorage-independent/soft-agar growth assays shown in Fig. 1E and 1F. (D – E) S100A4 downregulation was achieved with lentiviral vectors as shown in Fig. 1. S2VP10 and MIA PaCa-2 (MIA PaCa) cells with or without shRNA infection were serum starved with serum-free medium

(DMEM with 1% BSA) for 24 hours, followed by treatment with TGF-β1 (4 ng/ml) and pan-caspase inhibitor Z-VAD-fmk (20 μmol/L), or vehicle for 24 hours in serum-free medium. Annexin-V and PI labeling apoptosis assays were performed as described in Materials and Methods with S2VP10 and MIA PaCa-2 (MIA PaCa) cells, respectively. The percentage of early apoptotic cells were marked by Annexin-V-positive and PI-negative. Z-VAD-fmk treatment significantly reduced the percentage of Annexin-positive and PI-negative cells, supporting the role of caspase-mediated apoptosis during the events. The experiments were repeated 3-4 times. Data are represented as mean + SE. * represents $p < 0.01$ for the indicated two groups.

Supplementary Figure S6.

Inhibition of both Src and FAK activation induces cleavage of caspase-3 and PARP, and induces apoptosis in pancreatic cancer cells

S2VP10 cells were treated with Src inhibitor (Dasatinib), FAK inhibitor (PF-573228) or both at indicated dose. (A) Cells were lysed and equivalent amounts of whole tissue lysates were Western blotted with indicated antibodies. The antibodies specifically recognize the cleaved Caspase-3 and PARP and do not recognize the full length forms. (B) Annexin-V and PI labeling apoptosis assays were performed as described in Materials and Methods. The percentage of early apoptotic cells were marked by Annexin-V-positive and PI-negative. The

experiments were repeated 3-4 times. Data are represented as mean + SE. * represents $p < 0.01$ for the indicated two groups.

Supplementary Figure S7.

S100A4 downregulation sensitizes MIA PaCa-2 to TGF-β1-induced cell growth inhibition and apoptosis

MIA PaCa-2 (MIA PaCa) pancreatic cancer cells infected with S100A4 or control shRNA were treated with or without TGF-β1 as described in Figure 2. Cells were lysed, and equivalent amount of lysates were Western blotted with indicated antibodies. Numbers below images represent densitometry of band intensity. The antibodies specifically recognize the cleaved Caspase-3 and PARP and do not recognize the full length forms.
